# 3-Benzyl-5-bromo­pyrazin-2(1*H*)-one

**DOI:** 10.1107/S1600536808003073

**Published:** 2008-02-06

**Authors:** Jo Alen, Liliana Dobrzańska

**Affiliations:** a Molecular Design & Synthesis, Department of Chemistry, Katholieke Universiteit Leuven, Celestijnenlaan 200F, B-3001 Leuven, Belgium; b Department of Chemistry, University of Stellenbosch, Private Bag X1, Matieland, South Africa

## Abstract

In the title compound, C_11_H_9_BrN_2_O, the mol­ecules are linked into *R*_2_^2^(8) dimers by paired N—H⋯O hydrogen bonds and these dimers are further stacked into columns along the *c* axis by π–π inter­actions between pyrazinone rings [centroid–centroid distance = 3.544 Å; the dihedral angle between the planes of these rings is 7.51 (16)°]. The title compound is a precursor for agents with potential use as pharmaceuticals.

## Related literature

For related literature, see: Betancur *et al.* (1997 ▶); Harrison *et al.* (1994 ▶); Rombouts *et al.* (2001 ▶, 2003 ▶); Snider *et al.* (1991 ▶).
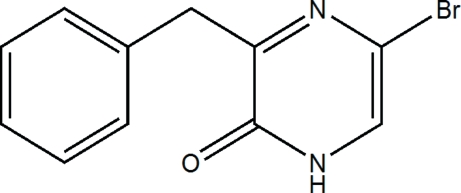


## Experimental

### 

#### Crystal data

C_11_H_9_BrN_2_O
*M* *_r_* = 265.11
Orthorhombic, 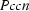

*a* = 12.0408 (16) Å
*b* = 24.273 (3) Å
*c* = 7.0428 (10) Å
*V* = 2058.4 (5) Å^3^
*Z* = 8
Mo *K*α radiation
μ = 3.97 mm^−1^
*T* = 100 (2) K
0.28 × 0.16 × 0.14 mm

#### Data collection

Bruker APEX CCD area-detector diffractometer
Absorption correction: multi-scan (*SADABS*; Sheldrick, 1997 ▶) *T*_min_ = 0.383, *T*_max_ = 0.576
9901 measured reflections
1825 independent reflections
1242 reflections with *I* > 2σ(*I*)
*R*_int_ = 0.097

#### Refinement

*R*[*F* ^2^ > 2σ(*F* ^2^)] = 0.051
*wR*(*F* ^2^) = 0.120
*S* = 0.99
1825 reflections
136 parameters
H-atom parameters constrained
Δρ_max_ = 0.71 e Å^−3^
Δρ_min_ = −0.55 e Å^−3^

### 

Data collection: *SMART* (Bruker, 2001 ▶); cell refinement: *SAINT* (Bruker, 2002 ▶); data reduction: *SAINT*; program(s) used to solve structure: *SHELXS97* (Sheldrick, 2008 ▶); program(s) used to refine structure: *SHELXL97* (Sheldrick, 2008 ▶); molecular graphics: *X-SEED* (Barbour, 2001 ▶; Atwood & Barbour, 2003 ▶); software used to prepare material for publication: *X-SEED*;.

## Supplementary Material

Crystal structure: contains datablocks I, global. DOI: 10.1107/S1600536808003073/kp2155sup1.cif

Structure factors: contains datablocks I. DOI: 10.1107/S1600536808003073/kp2155Isup2.hkl

Additional supplementary materials:  crystallographic information; 3D view; checkCIF report

## Figures and Tables

**Table 1:** Hydrogen-bond geometry (Å, °)

*D*—H⋯*A*	*D*—H	H⋯*A*	*D*⋯*A*	*D*—H⋯*A*
N3—H3⋯O8^i^	0.88	1.88	2.760 (5)	171

Symmetry code: (i) 
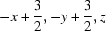
.

**Fig. 1.**
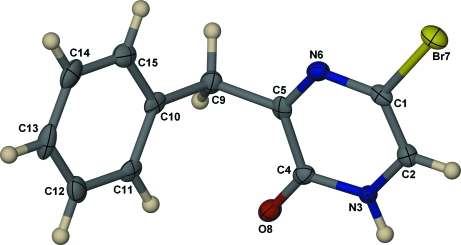
The molecular structure of (I) with the atom-numbering scheme. Displacement ellipsoids are drawn at the 50% probability level. H atoms are shown as spheres of arbitrary radii.

**Fig. 2.**
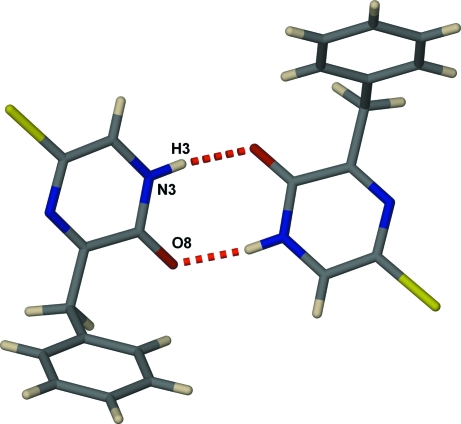
Hydrogen-bonded dimer. Hydrogen bonds are shown as dashed lines. The unlabeled molecule is related to the labeled one by the symmetry operation 3/2 - x, 3/2 - y, z.

**Fig. 3.**
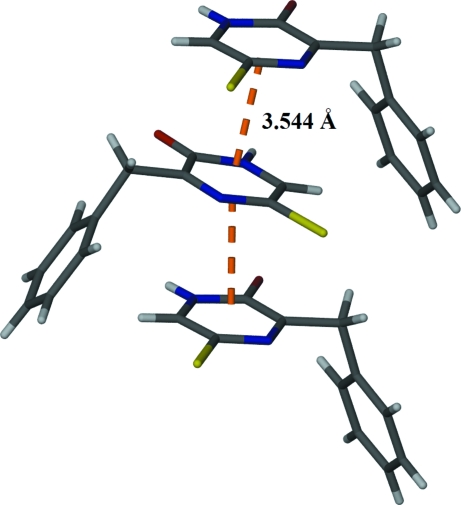
Capped-stick representation showing the π-π stacking geometry of (I) (dashed orange lines).

**Fig. 4.**
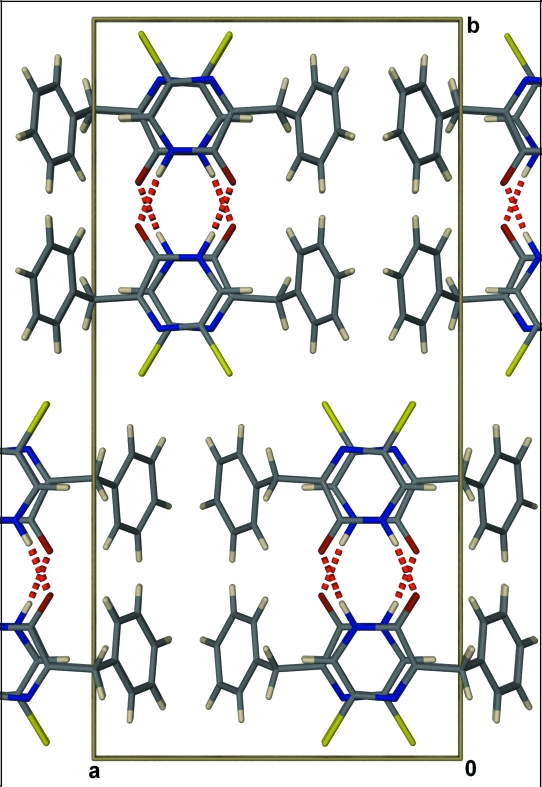
The packing of (I) viewed down [001].

## supplementary crystallographic information

### Comment

During the early nineties Pfizer (Snider *et al.*, 1991) and Merck (Harrison *et al.*, 1994) optimized a type of compounds (Betancur *et al.*, 1997) that may be of therapeutic use in the treatment of chronic pain, inflammation, depression, emesis, and asthma. (I) can be converted into similar agents with potential biological activity (Rombouts *et al.*, 2001; Rombouts *et al.*, 2003). The molecular structure is given in Fig. 1. The dihedral angle between the planes of the benzene ring (C10—C15) and the pyrazinone ring (C1—N6) is 67.1 (2)°. The r.m.s deviation from the mean plane for the C10—C15 benzene ring is 0.004 Å [maximum deviation = 0.007 (4) Å for atom C13]. For the pyrazinone ring the corresponding value is 0.009 Å [maximum deviation = 0.015 (4) Å for atom C5]. In the crystal packing around a twofold axes hydrogen-bonded dimers are formed through N3—*H*···O8^i^hydrogen bond [symmetry code: (i) 3/2 - *x*, 3/2 - *y*, *z*; distance of 2.760 (5) Å (Table 1, Fig. 2). These dimers are stacked into columns by π-π interactions between pyrazinone rings along the *c* axis [centroid···centroid distances = 3.544 Å; symmetry codes: (ii) 3/2 - *x*, *y*, 1/2 + *z* and (iii) 3/2 - *x*, *y*, -1/2 + *z*] (Fig. 3). There are no direction-specific interactions between stacked columns (Fig. 4).

### Experimental

105 mg (0.6 mmol) *N*-bromosuccinimide was added to an ice-cooled solution (273 K) of 100 mg (0.5 mmol) 3-benzyl-2(1*H*)-pyrazinone in anhydrous DMF and the mixture was stirred for 2 h at 273 K under inert atmosphere. After extraction with dichloromethane (3x), the organic layer was washed with water, dried over magnesium sulfate and concentrated *in vacuo*. The crude residue was purified by HPLC (column: Bio-Sil D90–10/250x10mm; Ref 614–0183; eluens: DCM/EtOAc 85:15; flow rate: 3 mL/min) to afford the desired product in 74% yield. IR (KBr, cm^-1^): 1640.9 (C=O), 1583.4 (C=N); ^1^H-NMR (300 MHz, CDCl_3_): 7.5–7.1 (m, 7H, NH + ArH), 4.1 (s, 2H, CH_2_); ^13^C-NMR (75 MHz, CDCl_3_): 136.3 (CO), 129.4–129.3–128.8–128.5–127.8–126.8 (9ArC), 39.3 (CH_2_); m/*z* (E.I., %): 264 (*M*^+^, 81), 263 (*M*^+^ - H, 62), 206 (C_8_H_15_OBr, 100), 185 (C_4_H_3_ON_2_Br, 74); HRMS (E.I.): exact mass calcd for C_11_H_9_N_2_OBr: 263.98982; found: 263.99082.

### Refinement

H atoms were positioned geometrically (C—H = 0.95 and 0.99 Å; N—H = 0.99 Å) and constrained to ride on their parent atoms; *U*_iso_(H) values were fixed at 1.2 times *U*_eq_(C).

### Crystal data

**Table:**

C_11_H_9_BrN_2_O	*D*_x_ = 1.711 Mg m^−^^3^
*M**_r_* = 265.11	Melting point: 428 K
Orthorhombic, *P**c**c**n*	Mo *K*α radiation λ = 0.71073 Å
Hall symbol: -P 2ab 2ac	Cell parameters from 862 reflections
*a* = 12.0408 (16) Å	θ = 3.0–18.1º
*b* = 24.273 (3) Å	µ = 3.97 mm^−^^1^
*c* = 7.0428 (10) Å	*T* = 100 (2) K
*V* = 2058.4 (5) Å^3^	Block, pale yellow
*Z* = 8	0.28 × 0.16 × 0.14 mm
*F*_000_ = 1056	

### Data collection

**Table:**

Bruker APEX CCD area-detector diffractometer	1825 independent reflections
Radiation source: fine-focus sealed tube	1242 reflections with *I* > 2σ(*I*)
Monochromator: graphite	*R*_int_ = 0.097
*T* = 100(2) K	θ_max_ = 25.0º
ω scans	θ_min_ = 1.7º
Absorption correction: multi-scan(SADABS; Sheldrick, 1997)	*h* = −14→14
*T*_min_ = 0.383, *T*_max_ = 0.576	*k* = −28→28
9901 measured reflections	*l* = −8→6

### Refinement

**Table:**

Refinement on *F*^2^	Secondary atom site location: difference Fourier map
Least-squares matrix: full	Hydrogen site location: inferred from neighbouring sites
*R*[*F*^2^ > 2σ(*F*^2^)] = 0.051	H-atom parameters constrained
*wR*(*F*^2^) = 0.120	*w* = 1/[σ^2^(*F*_o_^2^) + (0.0603*P*)^2^] where *P* = (*F*_o_^2^ + 2*F*_c_^2^)/3
*S* = 1.00	(Δ/σ)_max_ = 0.001
1825 reflections	Δρ_max_ = 0.71 e Å^−^^3^
136 parameters	Δρ_min_ = −0.55 e Å^−^^3^
Primary atom site location: structure-invariant direct methods	Extinction correction: none

### Special details

**Table:**

Geometry. All e.s.d.'s (except the e.s.d. in the dihedral angle between two l.s. planes) are estimated using the full covariance matrix. The cell e.s.d.'s are taken into account individually in the estimation of e.s.d.'s in distances, angles and torsion angles; correlations between e.s.d.'s in cell parameters are only used when they are defined by crystal symmetry. An approximate (isotropic) treatment of cell e.s.d.'s is used for estimating e.s.d.'s involving l.s. planes.
Refinement. Refinement of *F*^2^ against ALL reflections. The weighted *R*-factor *wR* and goodness of fit *S* are based on *F*^2^, conventional *R*-factors *R* are based on *F*, with *F* set to zero for negative *F*^2^. The threshold expression of *F*^2^ > σ(*F*^2^) is used only for calculating *R*-factors(gt) *etc*. and is not relevant to the choice of reflections for refinement. *R*-factors based on *F*^2^ are statistically about twice as large as those based on *F*, and *R*- factors based on ALL data will be even larger.

### Fractional atomic coordinates and isotropic or equivalent isotropic displacement parameters (Å^2^)

**Table:**

	*x*	*y*	*z*	*U*_iso_*/*U*_eq_	
C1	0.7911 (5)	0.5858 (2)	0.1629 (8)	0.0200 (13)	
C2	0.8466 (4)	0.6322 (2)	0.1986 (9)	0.0224 (13)	
H2	0.9253	0.6335	0.1908	0.027*	
N3	0.7866 (3)	0.67783 (18)	0.2467 (6)	0.0203 (11)	
H3	0.8225	0.7086	0.2708	0.024*	
C4	0.6734 (5)	0.6778 (2)	0.2591 (8)	0.0210 (13)	
C5	0.6231 (4)	0.6248 (2)	0.2201 (7)	0.0210 (12)	
N6	0.6796 (4)	0.58141 (18)	0.1693 (7)	0.0224 (11)	
Br7	0.86975 (5)	0.52084 (2)	0.10346 (9)	0.0303 (2)	
O8	0.6208 (3)	0.71960 (14)	0.3066 (6)	0.0260 (9)	
C9	0.4979 (4)	0.6209 (2)	0.2288 (8)	0.0248 (14)	
H9A	0.4697	0.6479	0.3223	0.030*	
H9B	0.4768	0.5836	0.2734	0.030*	
C10	0.4439 (4)	0.6316 (2)	0.0387 (8)	0.0207 (13)	
C11	0.4358 (4)	0.6843 (2)	−0.0314 (8)	0.0217 (13)	
H11	0.4676	0.7141	0.0374	0.026*	
C12	0.3819 (4)	0.6943 (2)	−0.2013 (9)	0.0299 (14)	
H12	0.3773	0.7309	−0.2487	0.036*	
C13	0.3350 (5)	0.6521 (3)	−0.3016 (10)	0.0344 (16)	
H13	0.2966	0.6594	−0.4168	0.041*	
C14	0.3437 (4)	0.5990 (3)	−0.2346 (9)	0.0314 (16)	
H14	0.3126	0.5694	−0.3052	0.038*	
C15	0.3968 (4)	0.5887 (2)	−0.0673 (9)	0.0268 (15)	
H15	0.4020	0.5519	−0.0220	0.032*	

### Atomic displacement parameters (Å^2^)

**Table:**

	*U*^11^	*U*^22^	*U*^33^	*U*^12^	*U*^13^	*U*^23^
C1	0.027 (3)	0.017 (3)	0.015 (3)	0.004 (2)	−0.002 (2)	−0.002 (2)
C2	0.021 (3)	0.024 (3)	0.022 (3)	0.003 (3)	−0.002 (3)	0.001 (3)
N3	0.016 (2)	0.016 (2)	0.028 (3)	−0.001 (2)	−0.003 (2)	0.004 (2)
C4	0.023 (3)	0.022 (3)	0.018 (3)	−0.004 (3)	−0.002 (2)	0.004 (3)
C5	0.021 (3)	0.031 (3)	0.011 (3)	0.001 (3)	−0.001 (3)	0.008 (2)
N6	0.025 (3)	0.021 (3)	0.021 (3)	−0.004 (2)	−0.003 (2)	0.004 (2)
Br7	0.0358 (4)	0.0211 (3)	0.0341 (4)	0.0062 (3)	0.0004 (3)	−0.0002 (3)
O8	0.022 (2)	0.024 (2)	0.033 (2)	−0.0024 (18)	0.0019 (19)	−0.0004 (19)
C9	0.022 (3)	0.031 (3)	0.021 (3)	−0.003 (3)	0.000 (3)	0.007 (3)
C10	0.013 (3)	0.026 (3)	0.023 (3)	−0.001 (2)	0.011 (2)	−0.003 (3)
C11	0.013 (3)	0.025 (3)	0.027 (3)	−0.002 (2)	0.012 (3)	−0.003 (3)
C12	0.017 (3)	0.036 (3)	0.037 (4)	0.004 (3)	0.001 (3)	0.009 (3)
C13	0.016 (3)	0.069 (5)	0.018 (3)	−0.006 (3)	−0.002 (3)	0.006 (4)
C14	0.014 (3)	0.043 (4)	0.037 (4)	−0.004 (3)	0.006 (3)	−0.007 (3)
C15	0.019 (3)	0.024 (3)	0.037 (4)	0.004 (3)	0.009 (3)	−0.002 (3)

### Geometric parameters (Å, °)

**Table:**

C1—C2	1.334 (7)	C9—H9B	0.9900
C1—N6	1.347 (7)	C10—C11	1.375 (7)
C1—Br7	1.886 (5)	C10—C15	1.401 (8)
C2—N3	1.365 (6)	C11—C12	1.383 (8)
C2—H2	0.9500	C11—H11	0.9500
N3—C4	1.366 (6)	C12—C13	1.366 (8)
N3—H3	0.8800	C12—H12	0.9500
C4—O8	1.243 (6)	C13—C14	1.378 (9)
C4—C5	1.447 (8)	C13—H13	0.9500
C5—N6	1.305 (7)	C14—C15	1.364 (9)
C5—C9	1.512 (7)	C14—H14	0.9500
C9—C10	1.512 (8)	C15—H15	0.9500
C9—H9A	0.9900		
			
C2—C1—N6	124.0 (5)	C10—C9—H9B	109.1
C2—C1—Br7	119.7 (4)	H9A—C9—H9B	107.8
N6—C1—Br7	116.2 (4)	C11—C10—C15	118.2 (6)
C1—C2—N3	117.8 (5)	C11—C10—C9	120.5 (5)
C1—C2—H2	121.1	C15—C10—C9	121.2 (5)
N3—C2—H2	121.1	C10—C11—C12	120.5 (6)
C2—N3—C4	122.9 (5)	C10—C11—H11	119.8
C2—N3—H3	118.6	C12—C11—H11	119.8
C4—N3—H3	118.6	C13—C12—C11	120.7 (6)
O8—C4—N3	121.7 (5)	C13—C12—H12	119.7
O8—C4—C5	124.3 (5)	C11—C12—H12	119.7
N3—C4—C5	114.0 (5)	C12—C13—C14	119.5 (6)
N6—C5—C4	123.4 (5)	C12—C13—H13	120.2
N6—C5—C9	118.7 (5)	C14—C13—H13	120.2
C4—C5—C9	117.8 (5)	C15—C14—C13	120.2 (6)
C5—N6—C1	117.7 (5)	C15—C14—H14	119.9
C5—C9—C10	112.5 (4)	C13—C14—H14	119.9
C5—C9—H9A	109.1	C14—C15—C10	120.9 (6)
C10—C9—H9A	109.1	C14—C15—H15	119.6
C5—C9—H9B	109.1	C10—C15—H15	119.6
			
N6—C1—C2—N3	0.8 (9)	N6—C5—C9—C10	−85.6 (6)
Br7—C1—C2—N3	−178.0 (4)	C4—C5—C9—C10	91.2 (6)
C1—C2—N3—C4	−0.2 (8)	C5—C9—C10—C11	−75.6 (6)
C2—N3—C4—O8	178.7 (5)	C5—C9—C10—C15	107.0 (6)
C2—N3—C4—C5	1.1 (7)	C15—C10—C11—C12	0.5 (8)
O8—C4—C5—N6	179.6 (5)	C9—C10—C11—C12	−177.0 (5)
N3—C4—C5—N6	−2.9 (8)	C10—C11—C12—C13	0.5 (8)
O8—C4—C5—C9	3.0 (8)	C11—C12—C13—C14	−1.4 (9)
N3—C4—C5—C9	−179.5 (5)	C12—C13—C14—C15	1.3 (9)
C4—C5—N6—C1	3.6 (8)	C13—C14—C15—C10	−0.4 (8)
C9—C5—N6—C1	−179.8 (5)	C11—C10—C15—C14	−0.5 (8)
C2—C1—N6—C5	−2.5 (9)	C9—C10—C15—C14	177.0 (5)
Br7—C1—N6—C5	176.4 (4)		

### Hydrogen-bond geometry (Å, °)

**Table:**

*D*—H···*A*	*D*—H	H···*A*	*D*···*A*	*D*—H···*A*
N3—H3···O8^i^	0.88	1.88	2.760 (5)	171

Symmetry codes: (i) −*x*+3/2, −*y*+3/2, *z*.

## References

[bb1] Atwood, J. L. & Barbour, L. J. (2003). *Cryst. Growth Des.* **3**, 3–8.

[bb2] Barbour, L. J. (2001). *J. Supramol. Chem.* **1**, 189–191.

[bb3] Betancur, C., Azzi, M. & Rostène, W. (1997). *Trends Pharmacol. Sci.* **18**, 372–386. 10.1016/s0165-6147(97)01109-7 9357322

[bb4] Bruker (2001). *SMART* Bruker AXS Inc., Madison, Wisconsin, USA.

[bb5] Bruker (2002). *SAINT* Bruker AXS Inc., Madison, Wisconsin, USA.

[bb6] Harrison, T., Williams, B. J. & Swain, C. J. (1994). *Bioorg. Med. Chem. Lett.* **4**, 2733–2734.

[bb7] Rombouts, F. J. R., De Borggraeve, W. M., Toppet, S. M., Compernolle, F. & Hoornaert, G. J. (2001). *Tetrahedron Lett.* **42**, 7397–7399.

[bb8] Rombouts, F. J. R., Van den Bossche, J., Toppet, S. M., Compernolle, F. & Hoornaert, G. J. (2003). *Tetrahedron*, **59**, 4721–4731.

[bb9] Sheldrick, G. M. (1997). *SADABS* University of Göttingen, Germany.

[bb10] Sheldrick, G. M. (2008). *Acta Cryst.* A**64**, 112–122. 10.1107/S0108767307043930 18156677

[bb11] Snider, R. M., Constantine, J. W., Lowe, J. A. III, Longo, K. P., Lebel, W. S., Woody, H. A., Drozda, S. E., Desai, M. C., Vinick, F. J., Spencer, R. W. & Hess, H.-J. (1991). *Science*, **251**, 435–437. 10.1126/science.1703323 1703323

